# Comparative analysis of thermoplastic masks versus vacuum cushions in stereotactic body radiotherapy

**DOI:** 10.1186/s13014-015-0484-7

**Published:** 2015-08-20

**Authors:** Arturo Navarro-Martin, Jon Cacicedo, Olwen Leaman, Ismael Sancho, Elvira García, Valentin Navarro, Ferran Guedea

**Affiliations:** Radiation Oncology Department, Hospital Duran i Reynals (ICO) Avda. Gran Via de L’Hospitalet, 199-203 08908 Hospitalet de Llobregat, Barcelona, Spain; Radiation Oncology department at Cruces University Hospital, c/Plaza de Cruces s/n 48903, Barakaldo, Vizcaya (Basque Country) Spain; Medical Physicist Department, Hospital Duran i Reynals (ICO) Avda. Gran Via de L’Hospitalet, 199-203 08908 Hospitalet de Llobregat, Barcelona, Spain; Medical Research Unit, Hospital Duran i Reynals (ICO) Avda. Gran Via de L’Hospitalet, 199-203 08908 Hospitalet de Llobregat, Barcelona, Spain

## Abstract

**Background:**

To compare thermoplastic masks (TMP) and vacuum cushion system (VCS) to assess differences in interfraction set up accuracy in patients treated with stereotactic radiotherapy (SBRT) for oligometastatic lung cancer. Secondarily, to survey radiotherapy technologists to assess their satisfaction with the two systems.

**Methods:**

Retrospective study of patients treated with lung SBRT between 2008 to 2012 at our institution. Immobilization was performed for 73 treatment sessions (VCS = 40; TMP = 33). A total of 246 cone-beams were analysed. Patients considered ineligible for surgery with a life expectancy ≥6 months and performance status > 1 were included. Target lesion location was verified by cone beam computed tomography (CBCT) prior to each session, with displacements assessed by CBCT simulation prior to each treatment session. Couch shifts were registered prospectively in **vertical**, longitudinal, and latero-lateral directions to obtain Kernel coordinates (3D representation). Technologists were surveyed to assess their satisfaction with indexing, positioning, and learning curve of the two systems. Setup displacements were obtained in all patients for each treatment plan and for each session. To assess differences between the immobilization systems, a t-test (Welch) was performed.

**Results:**

Mean displacements for the TMP and VC systems, respectively, were as follows: session one, 0.64 cm vs 1.05 cm (*p* = 0.0002); session two, 0.49 cm vs 1.02 cm (*p* < 0.0001), and session three, 0.56 vs 0.97 cm (*p* = 0.0011). TMP resulted in significantly smaller shifts vs. VCS in all three treatment sessions. Technologists rated the learning curve, set up, and positioning more highly for TMP versus VCS.

**Conclusions:**

Due to the high doses and steep gradients in lung SBRT, accurate and reproducible inter-fraction set up is essential. We found that thermoplastic masks offers better reproducibility with significantly less interfractional set up displacement than vacuum cushions. Moreover, radiotherapy technologists rated the TMP system higher. Taken together, these two findings suggest that TMP may be preferable to VCS. However, more research is needed to determine both inter- and intrafraction error to identify the optimal immobilisation system for use in lung SBRT.

## Introduction

An emerging non-surgical approach to treating inoperable lung cancer is stereotactic body radiotherapy (SBRT) [1]. Early results are extremely promising, with 3-year local control rates approaching 98 %, locoregional control of 88 % and overall survival of 56 % [2], with low rates of G3 toxicity (3–5 %) [1]. However, lung SBRT is a highly complex technique that uses high-dose radiation. As a result, delivery precision is paramount to avoid harming adjacent critical organs while limiting the radiation to the target volume in the lung. Because the lung is in constant motion due to respiratory motion, numerous techniques have been developed to adjust for this movement, including immobilization to minimize respiratory motion, real-time tumour tracking, and respiratory gating to coincide with a particular phase of the respiratory cycle. However, an important disadvantage of these methods—with the exception of immobilization—is that they significantly increase treatment duration [3].

The use of an accurate, reproducible and comfortable immobilization device is essential, particularly because most SBRT patients tend to be fragile, have limited mobility, advanced age, and diverse comorbidities, all of which can affect their positioning ability [4]. Although various guidelines for SBRT have been published [3, 5], no clear standard approach for immobilization has emerged to date. Moreover, little attention has been paid to this crucial aspect of SBRT in lung cancer, with only a few published studies on this topic, none of which were randomized [6–8].

Two of the most common immobilization systems in use are the vacuum cushion (VC) system and thermoplastic masks (TMP). Given the paucity of published data on the relative efficacy of these two systems, we carried out the present comparative study to check for differences in inter-fraction set up accuracy in patients undergoing SBRT for oligometastatic lesions. Additionally, given the important role of radiotherapy technologists in patient positioning procedures, a secondary aim was to survey these professionals to assess their satisfaction with the two systems.

## Materials and methods

This was a retrospective study of patients who underwent SBRT to treat lung cancer from 2008 to 2012. A total of 73 treatments were performed and a total of 246 cone-beams were analyzed. Prior to performing SBRT, all cases were evaluated by the institutional lung tumour board and the advanced techniques committee. Only patients considered unsuitable for local therapy (i.e., surgery or radio-frequency ablation) and therefore referred for SBRT were included in this study. Only patients with a life expectancy ≥6 months and a performance status 0-1 were included. Patient characteristics and treatments are described in Table 1.

**Table 1:** Patients Characteristics and treatments

Variable					
Age	Mean, 68 yrs (r, 39-89)	n = 73		%	
Target Location	Right Upper Lobe	28		38.35	
	Left Upper Lobe	22		30.13	
	Right Lower Lobe	10		13.7	
	Left Lower Lobe	8		11	
	T12 vertebrae	1		1.37	
	Median Lobe	3		4.10	
	Sacrum	1		1.37	
Primary	Lung	62		85	
	Colorectal	8		11	
	Breast	2		2.7	
	Neuroendocrine	1		1.37	
Treatment	Dose	TMP	VCS	%	
	34Gy x 1fr	9	0	12.3	0
	18Gy x 3fr	9	35	12.3	48
	12.5Gy x 4fr	9	3	12.3	4
	16Gy x 1fr	2	0	2.7	0
	7.5Gy x 8fr	4	1	5.5	1.3
	10Gy x 5fr	0	1	0	1.3
	Total	33	40	45	54

TMP indicates thermoplastic masks; VCS, vacuum cushion system; fr, fraction

Patients received instructions about breath control techniques prior to image acquisition at the time of the computed tomography (CT)-4D planning. Patients were instructed to breathe in and out following the rhythm marked by an acoustic signal. Patients who showed the capacity to control their breath in this way underwent image acquisition on the second day.

**Fig. 1:**
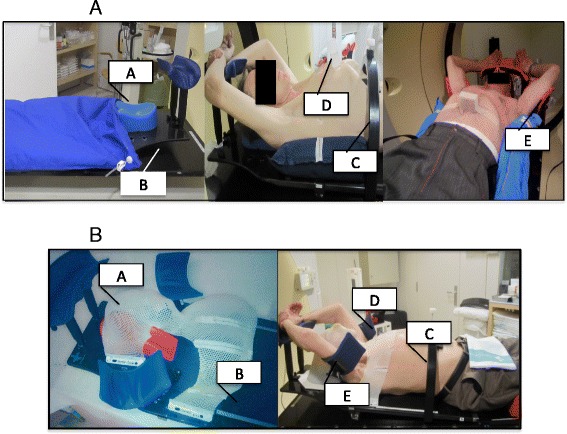
Thermoplastic mask and vacuum cushion system components. Fig. 1a depicts the vacuum cushion system with its components: **a**: Vacuum Cushion, **b**: Common Couch, **c**: Abdominal Compressor. **d**: Marker Block, **e**: Arm Support. Fig 1B shows the thermoplastic mask system and its components: **a**: Thermoplastic Mask, **b**: Common Couch, **c**: Abdominal Compressor. **d**: Marker Block **e**: Arm support

Patients were immobilized with one of two different immobilization systems (Figs. 1a & b) , as follows: 1) whole body vacuum cushions (VC) (Civco Medical Solutions; Kalona, Indiana, USA) or 2) thermoplastic mask (TMP) system (Lorca Marin S.A., Spain). The VC cushions are custom-formed for positioning accuracy, and become rigid when vacuum is applied. The TMP immobilization system consists of a malleable thermoplastic mask that is fixed to the patient from the head to the upper thorax, while leaving the arms free to place in the fixation system. Our institution initially utilized only the VC system, however, over time we progressively switched to TMP immobilization because we observed unacceptably large set-up errors with the VC system. As Fig. 1 clearly shows, both systems share many of the same components, including the couch, abdominal compressor, marker block, and arm support.

Overall, a total of 73 treatment sessions were immobilized using either the VC system (40 procedures) or the TMP system (33 procedures) (Table 1). An abdominal compressor was used in lower lobe lesions (2 patients) treated with a single 34 Gy dose**.**

### Contouring

The lesion was delineated in phases of maximum and minimal craniocaudal, latero-lateral and anteroposterior movement. The internal gross target volume (iGTV) was determined and expanded by 0.6 cm isotropically to contour the planning target volume (PTV). Dose fractionation and total dose was decided according to the localization of the lesion and size of the target according to National Cancer Comprehensive Network (NCCN) guidelines for non-small cell lung cancer (NSCLC), v4.2014 [9].

### Dose calculation

Dose calculation was performed using the Anisotropic Analytical Algorithm (AAA), and the prescribed dose was sufficient to assure D95 = 100 %. Once planning was designed and verified by the treatment team, the patient was scheduled to start treatment.

### Image acquisition

**Fig. 2:**
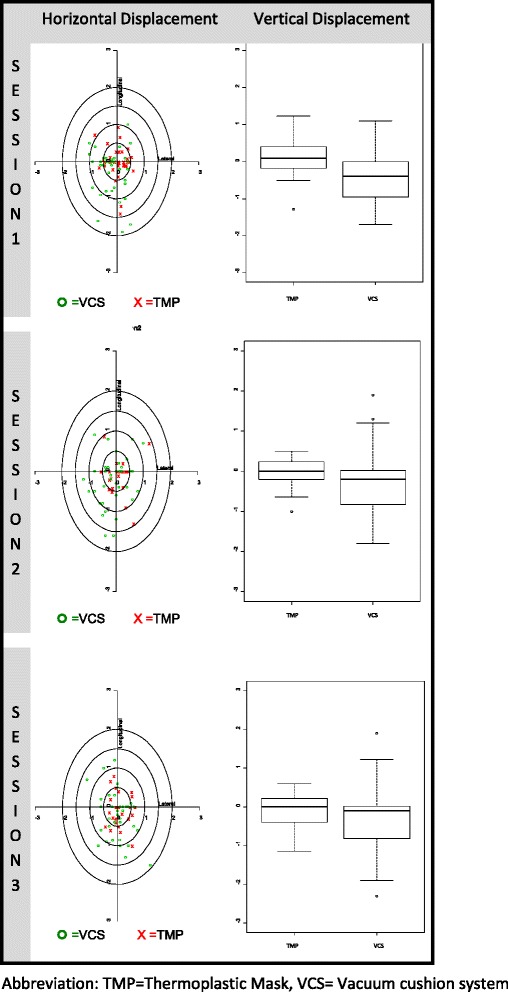
Plane-height graphics

Prior to each treatment session, target lesion location was verified by cone beam CT (CBCT). A radiation oncologist verified shifts in each treatment based on the CT simulation performed prior to the start the treatment session. Couch shifts were registered prospectively in **vertical**, longitudinal, and laterolateral directions for a total of 246 CBCTs (see Fig. 2)**.** These shifts were registered to TMP or VC to obtain Kernel coordinates, which allow us to create a 3D representation of the shifts and thus determine if there was an identifiable trend in either direction; we used a mathematical function of module (which provides a generalization of the notion of vector space over a field) to determine the magnitude of these shifts in all directions. A Welch’s test was performed to compare differences between magnitude of displacements (module) and variability in both immobilization systems using data from the first three sessions. Couch shifts after the fourth fraction were not eligible for analysis because the sample size of patients treated with VCS was insufficient.

### Technologist satisfaction survey

At our institution, three teams (2 technologists per team) are responsible for delivering SBRT treatments. To compare the two immobilization systems in terms of learning curve and reproducibility, the technologists completed a survey, the results of which are shown in Table 2. The survey was administered at the end of the study to ensure that all technologists had sufficient time and experience to thoroughly familiarize themselves with both immobilization systems. The survey included questions on indexing, positioning, and learning curve.

**Table 2:** Results of survey of technologists. Scores range from 1 (lowest) to 4 (highest)

	TMP	VC
Indexing	Mean Score	
Simple	2.86	1.61
Fast	2.43	1.61
Manageable	2.71	1.8
Mask Utility	3.86	NA
Additional Vacuum Cushion	1.43	NA
Arc Adjustment	3.14	2
Versatility	2.14	1.4
Positioning		
Simple	3.43	1.8
Fast	2.71	1.4
Reproducible	3.71	1
Comfortable	2.43	2.4
Learning Curve		
Simple	3.71	2.4
Fast	3.57	2.6

Abbreviation: *TMP* indicates thermoplastic ask; *VCS* vacuum cushion system; *NA* not applicable

#### Statistical analysis

Setup displacements were obtained in all patients for each treatment plan and for each session. In each session, displacements were initially obtained in the three dimensions (3D). Therefore, the shifts for each patient and session is expressed as a vector Si = (si1,si2,si3). If no displacement was registered for a given session, the shift was expressed as (0,0,0).

Several measurements were calculated to characterize displacement patterns:

- M_3D_ is the distance between the optimal point (0,0,0) and the displacement point considering three dimensions. M_3D_ is calculated using the classic mathematical formula of the module of a 3D vector.

$$ {M}_{3D}=\left\Vert Si\right\Vert =\sqrt{S_{i,1}^2+{S}_{i,2}^2+{S}_{i,3}^2} $$

-Plane-height graphics: Since this measure is difficult to represent graphically, displacements were separated into vertical and horizontal displacement and were shown in different graphical representations (see Fig. 2).

- ○ The vertical displacement measure is the s_i3_ component directly and indicates the elevation of the displacement.
- ○ For horizontal displacement, the *r* parameter was (also M_2D_) calculated as the distance between the optimum point (0,0) and the projection of the displacement point in the horizontal plane, which indicates the magnitude of the shift in the horizontal plane. This measure is obtained with the formula of the module but using only horizontal coordinates Si = (s_i1_,s_i2_) .

Numerical and graphical analyses were performed. Numerical analyses were performed for the M_3D_ measure, graphical analyses for horizontal shift using *r (or M*_*2D*_*)* and vertical shift using s_i3_. Box plot graphs show vertical, lateral and longitudinal displacement for the first three sessions. To assess differences between immobilization systems, a t-test (Welch) was performed based on the M_3D_measure and variability.

## Results

Displacements in the first three sessions are shown in Figure 2 using plane-height graphics. In this graph we found a significantly higher displacement in the vertical axes in VC versus TMP and a trend displacement to the right-lower region in the VC system compared to the TMP system.

**Fig. 3:**
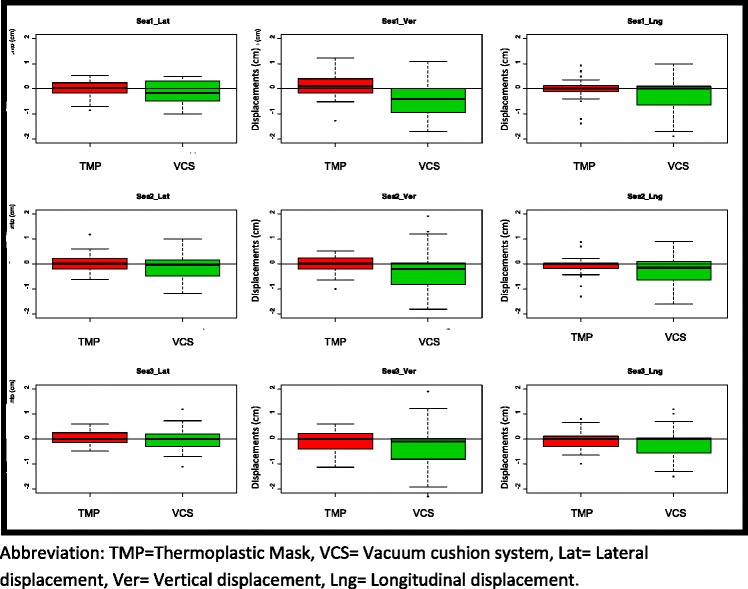
Displacements in Module

Box plots (Fig. 3) show shifts for **vertical**, lateral and longitudinal axis in the first three sessions for both immobilization systems. As the figure makes clear, displacement was significantly greater for VC vs. TMP.

Mean displacements (Table 3), measured in the M_3D_, for the TMP and VC systems, respectively, were as follows: session one, 0.64 cm vs 1.05 cm (*p* = 0.0002); session two, 0.49 cm vs 1.02 cm (*p* < 0.0001), and session three, 0.56 vs 0.97 cm (*p* = 0.0011). Thus, TMP resulted in significantly smaller shifts vs. VC in all three treatment sessions. Similarly, analysis of variance (ANOVA) revealed that VC presented a significantly greater ANOVA than TMP in both sessions 1 and 3 (Table 3).

**Table 3:** Mean Displacements and Variance

	MEAN		T- test Welch		Variance ANOVA test	
	TMP	VCS	Value	p-value	ratio (TMP/VCS)	p-value
Session 1	0.64	1.05	−3.91	0.0002	0.4007	0.0081
Session 2	0.49	1.02	−4.49	<0.0001	0.5921	0.1508
Session 3	0.56	0.97	−3.41	0.0011	0.3631	0.0075

Abbreviation: *TMP* Thermoplastic Mask, *VCS* Vacuum cushion system

Levels of satisfaction on the satisfaction survey were consistently lower for the VCS system vs. the TMP. Technologists considered that the learning curve, set up, and positioning were all more satisfactory in TMP than VCS (Fig. 3).

## Discussion

Considering the large dose fractions and steep gradients used in SBRT, accurate and reproducible set up between fractions is essential, particularly in lung cancer where critical organs and structures are located in close proximity to the target. In the present study, we have demonstrated that thermoplastic masks offers better and easier reproducibility and present significantly less interfractional set up displacement than vacuum cushions. Moreover, radiotherapy technologists find the TMP system to be more “user friendly”. Taken together, these two findings suggest that TMP may be preferable to VCS.

Determining the optimal immobilization system is a complex task given the wide variety of systems currently in use, some of which are custom-made. However, with the growth of SBRT and other high-dose radiotherapy modalities, the importance of accurate and reproducible patient set and immobilization is more important than ever. This is especially true in lung cancer due to organ motion and the presence of critical structures located adjacent to the target volume.

Various groups have published the results of their experience in terms of local control and toxicity using commercially available immobilization systems for SBRT [4, 6, 8]. Treatment guidelines for SBRT, including the EORTC recommendations for planning and delivery of high-dose, high-precision radiotherapy for lung cancer [3], include general recommendations about immobilization systems. The EORTC recommendations state that such systems should be both reproducible and safe, which is especially important for SBRT. These same guidelines also state that some studies have found that no immobilization system is necessary. Given this ambiguity, until more definitive guidelines or randomized controlled trials are published, each centre needs to select an approach based on the best-available evidence and the characteristic (resources, staff preferences, patient type) of the centre.

### Conventional or specific immobilization

In a cohort study of SBRT for pulmonary metastases, Siva et al. found that in addition to reducing tumour excursion and intrafraction error, the vacuum immobilisation facilitates reproducible positioning. However, an important limitation of that study was the small sample size (only 19 treatments) [6]. In contrast, Nielsen et al. [7] compared a standard fixation system to a custom-made system. These authors concluded that systematic and random setup uncertainties were the same, regardless of the different fixation equipment used. As a result, they conclude that margins cannot be reduced by changing fixating equipment. For these authors, the imaging protocol is a more important factor.

Sonke et al. [10] published their experience in 65 patients with small peripheral lung lesions treated without body frame to 54Gy in three fractions. They used an imaging protocol involving three 4D-CBCTs. One image was taken prior to treatment, the second was performed after corrections, with the final one at the end of the treatment. They concluded than SBRT treatment can be safely administered without a specific immobilization device when a three 4D-CBCT imaging protocol is used. Similarly, Dahele and colleagues [11] concluded that rigid external immobilization devices are not necessary in most cases of patients undergoing lung SBRT.

These data suggest that the choice of a specific immobilization system may not be essential because the imaging protocol may actually be more important. However this question remains unresolved. Some SBRT guidelines, such as those developed by Task Group 101 [5], state that although an imaging protocol can reduce the need for a proper immobilization system, it cannot eliminate it. Moreover, a drawback of relying on imaging is that such protocols are resource intense and require increased machine time. In addition, because many centres may not possess a 4D-CBCT, immobilization systems are crucial for such centres.

### Utility of abdominal compression in lung SBRT

Bouilhol et al. [12] performed a 4D-CT and dosimetric lobe-dependent study to determine the usefulness of abdominal compression in lung SBRT as a function of lobe tumor location. Those authors found that abdominal compression had the most significant impact on outcomes in patients with lower lobe tumors. In contrast, minor or negative effects were reported for patients with lesions located in other areas of the lung, and lung sparing was not substantially improved. Bengua and colleagues [13] reported a similar findings with regard to the benefits of abdominal compression in lower lung lesions. Finally, Richmond et al. [14] reported that abdominal compression led to a greater variation in set-up errors and changes in the mean value.

Based on these data, we can conclude that lesions located in the lower lobe are most likely to benefit the most from abdominal compression. At our institute, patients with upper or middle lobe lesions are now systematically treated without compression while the usefulness of compression for lower lobe tumors is considered on an individual basis.

### Differences between immobilization systems

The William Beaumont group [15] published a study of intrafraction variation (IFV) of mean tumour position during image-guided hypofractionated SBRT for lung cancer. The authors found that prolonged delivery times during daily CBCT-guided lung SBRT led to higher IFV of the mean target position (MTP). Significant differences in IFV-MTP were seen between immobilization devices. The stereotactic frame immobilization device was found to be significantly less likely to have an IFV-MTP vector > 2 mm compared to the alpha cradle, BodyFIX, and hybrid immobilization devices. The results of that study suggest that each immobilization system should be tested to determine setup errors and IFV. Although our study did not evaluate IFV, immobilization systems should also be tested to assess IFV, and a study to evaluate this setting is ongoing in our center.

Differences in the Body-Fix and abdominal compression plate (ACP) have been reported by Han et al. [8]. Those authors found no differences between the systems in IFV, but ACP was more comfortable, faster to set up, and presented lower superior-inferior shifts and less overall respiratory tumour motion than the Body-Fix.

### Satisfaction of radiotherapy technologists with the various immobilization systems

To our knowledge, none of the studies carried out to date to compare immobilization systems in lung cancer has attempted to determine the level of satisfaction of technologists. We believe this is an important and overlooked aspect of patient set up. The work of the technologist is an essential part of achieving correct patient positioning and, thereby, a lower systematic error. However, we must stress that while satisfaction of the technologists is important in choosing an immobilization system, it is less important than the system’s ability to deliver reliable, reproducible, and accurate patient set-up. Nevertheless, we believe that technologists’ preferences could have an impact on set-up accuracy. In addition, the fact that technologists prefer the TMP system, which we have shown to be significantly more accurate than the VC system in terms of displacement, adds additional support to help in choosing one system over another. The technologists’ preferences may, in part, be related to the patients’ performance status. Most of the patients included in this study were performance status 1 (PS = 1), and, as Li et al reported [4], such patients are more likely to drift out of position during SBRT treatment than PS 0 patients. Moreover, such patients are also less able to cooperate fully with technicians when using vacuum cushions. Similarly, the technologists’ perceived easier learning curve for thermoplastic masks may be because the masks used at our centre are customized versions of the head and neck masks which the technologists are already familiar with. As a result, the learning curve for the VC system was steeper because it was a new method.

Although we did not evaluate patient satisfaction and preferences in this study, this is obviously another important aspect to consider since patient comfort is important to reduce unwanted movement. This aspect will need to be evaluated in any future studies.

### Study limitations

An important limitation of this study is that we evaluated only interfractional displacement, but not interfractional shifts. Another limitation is that we primarily included lung SBRT. There may be important setup differences in different tumour locations, but we did not assess these as nearly all patients treated with SBRT at our centre are lung cancer patients. Finally, the sample size was relatively small (73 treatment fractions), and a larger sample would have provided more robust findings. The same holds true for the satisfaction survey, which involved only 6 technologists, thus making it difficult to reach any definitive conclusions. However this is the first paper to consider the opinion of technologists and for that reason this information is valuable. In addition, we did not survey the patients, even though this would have added valuable information to the study. Based on our experience with the present study, we now routinely ask for and record patient comfort levels.

## Conclusion

In general, there is a notable lack of evidence regarding the optimal immobilization systems for SBRT for lung cancer. We found that thermoplastic masks are more reliable than vacuum cushions and, moreover, are favoured by the technologists who are responsible for patient positioning. However, evidence in favour of one system or another remains relatively weak, and some authors even argue that no immobilization system is necessary. Ideally, large randomized studies that include a wider range of immobilisation devices are needed to determine both inter- and intrafraction error to identify the optimal immobilisation system for use in lung SBRT. Until then, however, system selection must be based on the available evidence.
